# Increasing incidence of rotator cuff repairs**—**A nationwide registry study in Finland

**DOI:** 10.1186/s12891-015-0639-6

**Published:** 2015-08-12

**Authors:** Juha Paloneva, Vesa Lepola, Ville Äärimaa, Antti Joukainen, Jari Ylinen, Ville M Mattila

**Affiliations:** Department of Surgery, Central Finland Hospital, Keskussairaalantie 19, 40620 Jyväskylä, Finland; Division of Orthopedics and Traumatology, Department of Trauma, Musculoskeletal Surgery and Rehabilitation, Tampere University Hospital, Tampere, Finland; Department of Orthopaedics and Traumatology, Turku University Hospital, Turku, Finland; Department of Orthopaedics, Traumatology and Hand Surgery, Kuopio University Hospital, Kuopio, Finland; Department of Physical and Rehabilitation Medicine, Central Finland Hospital, Jyväskylä, Finland; Department of Clinical Science, Intervention and Technology, Karolinska Institutet, Division of Orthopedics and Biotechnology, Karolinska Institutet and Department of Orthopedics at Karolinska University Hospital Huddinge, Sweden, and Department of Orthopedics, Tampere University Hospital, Tampere, Finland

## Abstract

**Background:**

Rotator cuff repair incidence rates have reportedly increased in the United States and England. Here we analyzed nationwide data relating to rotator cuff repairs recorded in the Finnish National Hospital Discharge Register (NHDR).

**Methods:**

The NHDR was reviewed to identify adult patients who underwent rotator cuff repair between 1998 and 2011. Incidence rates per 10^5^ person-years were calculated using the annual adult population size.

**Results:**

During the 14-year time period, 50,646 rotator cuff repairs were performed on subjects aged 18 years or older. The incidence of rotator cuff repair showed an almost linear increase of 204 %, from 44 per 10^5^ person-years in 1998 to 131 per 10^5^ person-years in 2011. The most common concomitant procedure was acromioplasty, which was performed in approximately 40 % of rotator cuff repairs in 2011. Other common concomitant procedures included tenodesis (7 %) and tenotomy (6 %) of the long head of the biceps tendon, and resection of the acromioclavicular joint (3 %).

**Conclusions:**

This nationwide analysis revealed a remarkable increase in the incidence of rotator cuff repair from 1998 to 2011 in Finland. This progress can be questioned, since there are not convincing data of the superiority of the operative treatment over non-operative management in all rotator cuff tears.

## Background

Rotator cuff tears are a leading cause of prolonged shoulder pain and disability but they may also be asymptomatic [1]. A tear is most commonly attributed to degeneration of the rotator cuff tendons, but may be associated also with a trauma [2, 3]. Non-traumatic rotator cuff tears were classically described as a continuum starting with acute tendon inflammation and developing into a full-thickness rotator cuff tear [4]. Nowadays, degenerative rotator cuff disease is considered to be of multifactorial etiology that includes both extrinsic and intrinsic mechanisms. Muscle imbalance and other functional deficiencies are also highlighted as predisposing factors [3, 5].

Rotator cuff disorders impose a substantial health-economic burden on individual patients and on society [1, 6]. Commonly advocated rotator cuff tear treatments include non-operative management, rotator cuff repair, and subacromial decompression (i.e., acromioplasty and bursa resection) [6–8]. Surgery can be performed using either an open or an arthroscopic approach [9, 10].

Incidences of rotator cuff repair have been reported to rapidly increase in selected patient cohorts [11, 12], but nationwide incidence rates remain unknown. The present study investigated the rotator cuff repair incidence rate in Finland over a 14-year time period. Based on previous reports, we hypothesized that the overall incidence of rotator cuff repair would show an increase over the time period.

## Patients and methods

### Design and setting

We reviewed nationwide data from the Finnish National Hospital Discharge Register (NHDR) for the time period 1998–2011. Founded in 1967, the NHDR contains data on age, sex, domicile, type of hospital (public or private), length of hospital stay, primary and secondary diagnosis, and operations performed during the hospital stay.

### Study population

This study included inhabitants of Finland aged 18 years or over. The operative treatment code NBL00 was used for both open and arthroscopic rotator cuff repair according to the Nomesco Classification of Surgical Procedures (Finnish version). This coding system did not allow us to differentiate between open and arthroscopic approaches used for rotator cuff repairs performed during the study period. Several procedure and diagnosis codes can be recorded for the same operation. Thus, we also examined concomitant procedures that were associated with rotator cuff repair (NBL00)—such as NBG10 for open acromioplasty, NBG15 for arthroscopic acromioplasty, NBL22 for tenotomy of the long head of the biceps tendon, NBL68 for tenodesis of the long head of the biceps tendon, and NBG00 for resection of the distal end of the clavicle.

We further investigated the ICD-10 diagnosis codes to estimate the cause of rotator cuff tear. Rotator cuff tear was considered non-traumatic if rotator cuff repair was performed in association with the diagnosis code M75.1 (rotator cuff disease) or M75.4 (subacromial impingement syndrome). The tear was deemed traumatic if tendon repair was associated with the code S46.0 (traumatic rotator cuff tear).

### Statistics

The primary outcome variable in this study was the incidence of rotator cuff repair per 10^5^ person-years, which was analyzed with stratification by age, sex, and study year. The secondary outcome variables were the etiology of rotator cuff tear according to ICD-10 diagnosis, and the percentage of concomitant procedures associated with tendon repair. Incidence rates were calculated using the annual adult population size (ranging from 4 million to 4.3 million during the study period) obtained from Statistics Finland, a statutory electronic national population register [13]. The incidence was based on the size of the entire population of persons ≥18 years old in Finland rather than on cohort-based estimates. Accordingly, confidence intervals were not calculated. Statistical analyses were performed using SPSS 22.0 software.

### Ethics

Ethics approval was granted by Finland’s National Institute for Health and Wellness (Dnr THL/89/5.05.00/2012).

## Results

During the 14-year study period, 50,646 rotator cuff repairs were performed on subjects aged 18 years and older in Finland. Over the study period, the mean patient age at the time of operation increased from 55 (SD 9) years in 1998 to 56 (SD 10) years in 2011. Among men, this mean age changed from 54 (SD 9) to 56 (SD 10), while the mean age among women changed from 57 (SD 9) to 59 (SD 9). The median length of hospital stay was 1 day (range, 0–49 days).

**Fig. 1:**
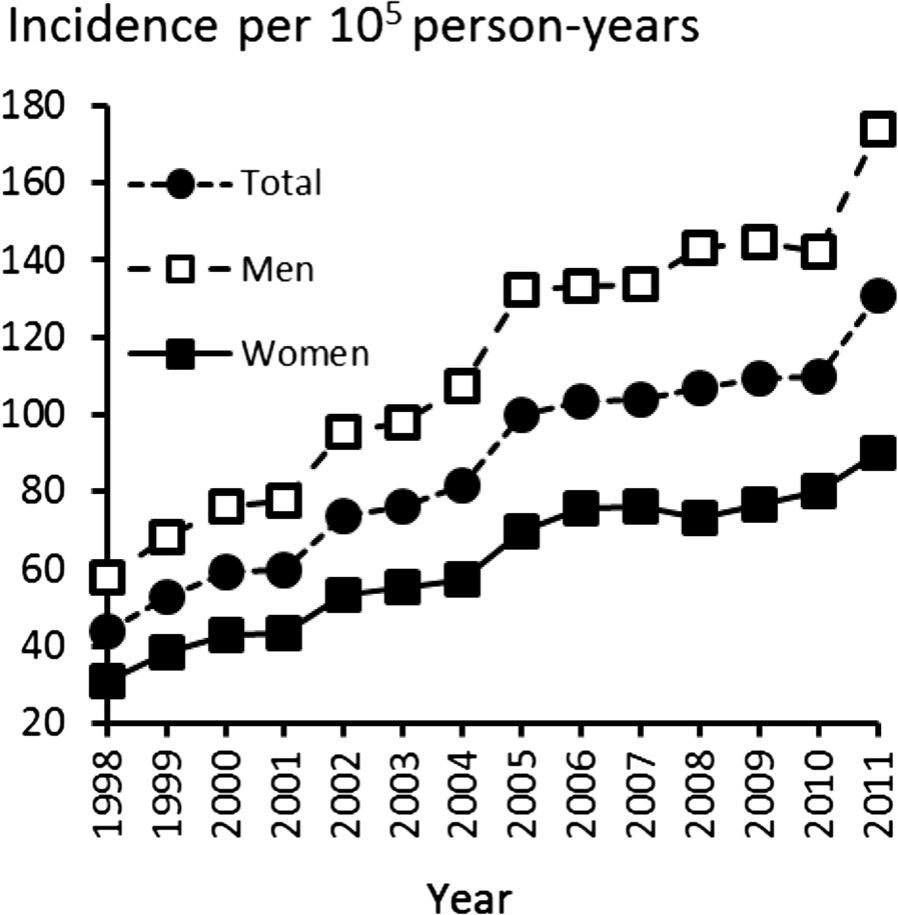
Incidence rates of rotator cuff repair in Finland by year

**Fig. 2:**
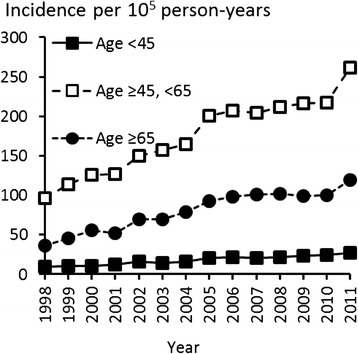
Incidence rates of rotator cuff repair among patients in different age groups in Finland by year

Figure 1 shows the incidence of rotator cuff repair for each year during the study period. From 1998 to 2011, the incidence of operations increased from 44 to 131 per 10^5^ person-years. The corresponding increases in incidence rate were 58 to 174 per 10^5^ person-years among men, and 31 to 90 per 10^5^ person-years among women. The male to female ratio was 1.7:1 (Fig. 1). The rise in incidence was most prominent within the middle-aged population (age 45–64 years), where an increase from 97 to 261 operations per 10^5^ person-years was observed. Similar but less dramatic trends were observed in the groups of patients who were 18–44 years old and over 65 years old (Fig. 2).

**Fig. 3:**
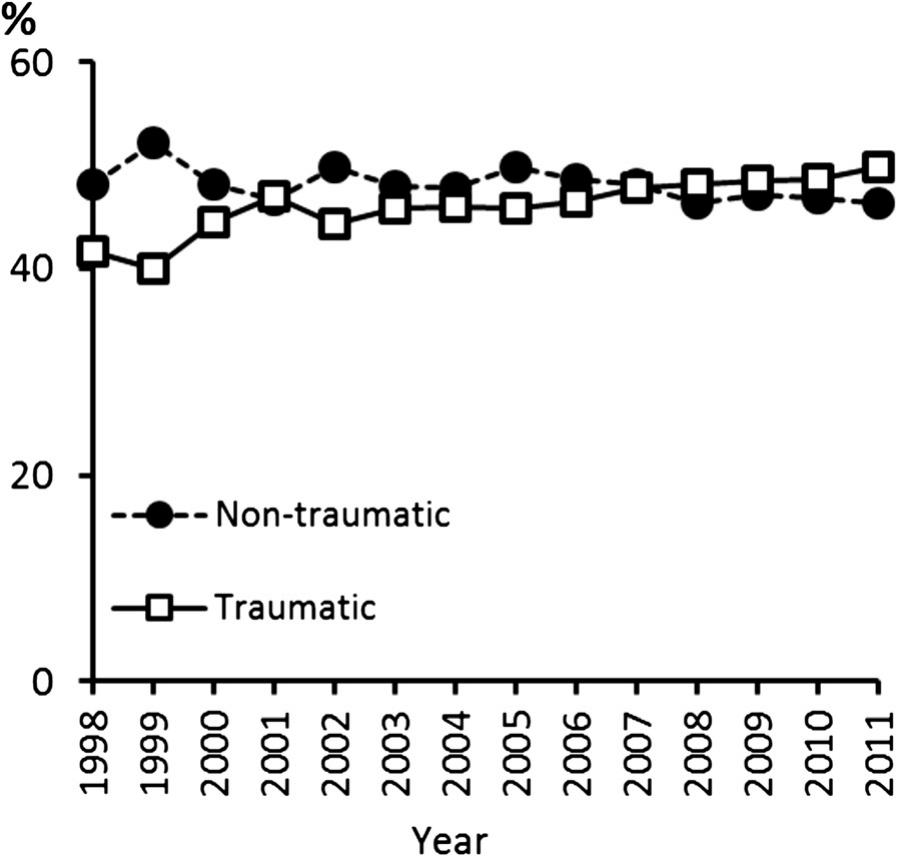
Etiology of rotator cuff tears. The repaired tear was deemed non-traumatic if the associated ICD-10 diagnosis code was M75.1 or M75.4. The tear was considered to be caused by trauma if the repair was associated with the ICD-10 code S46.0

Diagnosis codes indicated that the rotator cuff tear was interpreted non-traumatic in 48 % of cases and traumatic in 47 % of cases. The remaining 5 % included various diagnosis codes. These percentages remained at the same levels throughout the study period (Fig. 3).

**Fig. 4:**
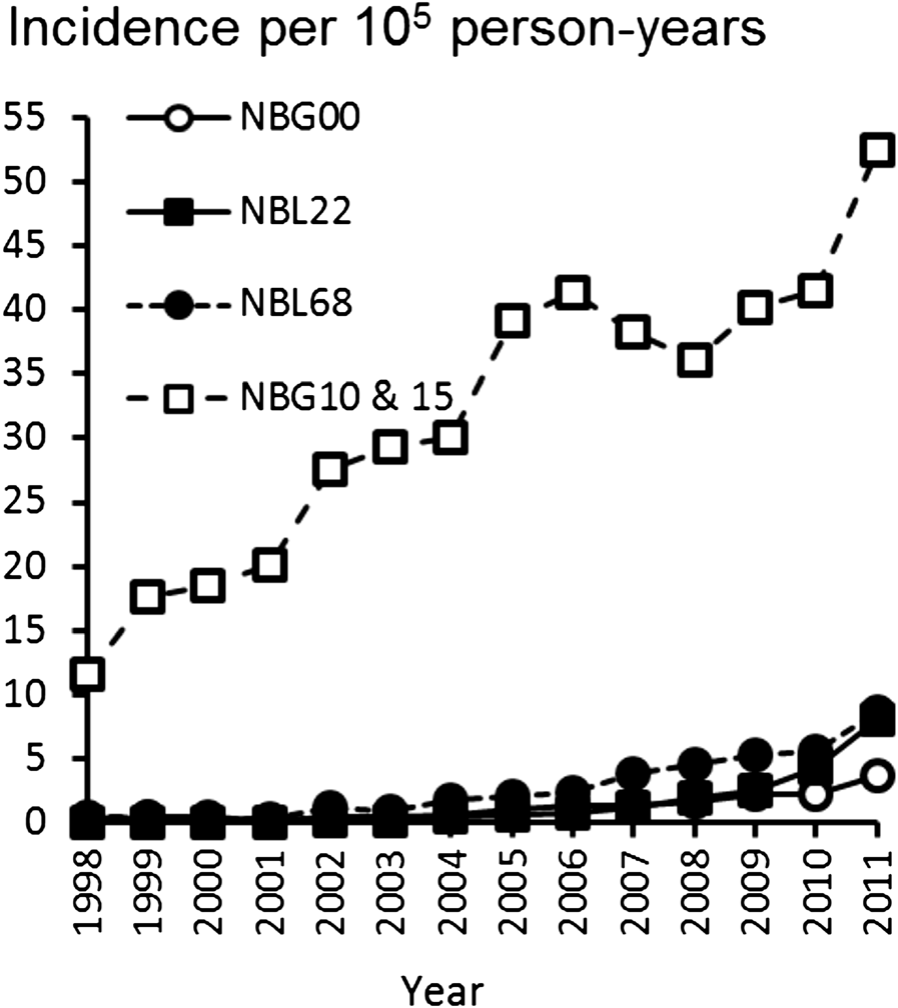
Incidence rates of concomitant procedures associated with rotator cuff repair in Finland by year. NBG10, open acromioplasty; NBG15, arthroscopic acromioplasty; NBL22, tenotomy of long head of biceps; NBL68, tenodesis of long head of biceps; NBG00, resection of the acromioclavicular joint

The most common concomitant procedure associated with rotator cuff repair was acromioplasty, which was performed in 26 % of operations in 1998 and in 40 % of the operations in 2011. Other common concomitant procedures included tenotomy of the long head of the biceps tendon, tenodesis of the long head of the biceps tendon, and resection of the acromioclavicular joint. From 1998 to 2011, the proportions of these procedures, respectively, increased from 0.1 % to 6 %, 1 % to 7 %, and 1 % to 3 % of the rotator cuff repairs. The incidences of concomitant procedures have been presented in Fig. 4.

**Fig. 5:**
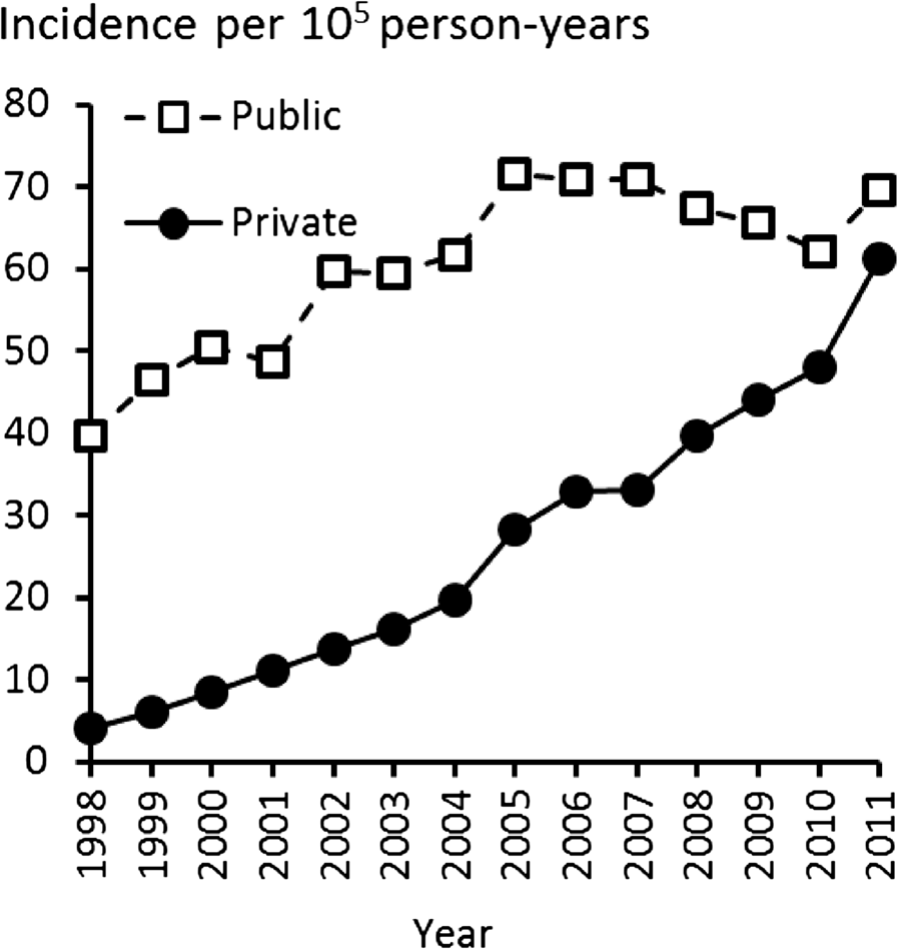
Rotator cuff repair incidence rates in public and private hospitals in Finland by year

From 1998 to 2005, the incidence rate of rotator cuff repair in public hospitals increased from 40 to 71 per 10^5^ person-years, and remained at the higher level thereafter until 2011. The incidence in private hospitals increased almost exponentially during the study period, rising by 1400 %, from 4 to 61 operations per 10^5^ person-years (Fig. 5). In 1998, 91 % of rotator cuff repairs were performed in public hospitals and 9 % in private hospitals. In 2011, the corresponding percentages were 53 % and 47 %.

## Discussion

The main finding of our present study was that the overall nationwide incidence of rotator cuff repair tripled between 1998 and 2011, confirming our hypothesis that the rotator cuff repair incidence had increased.

### Strengths and weaknesses

Strengths of this study included the use of reliable nationwide data and that the study population comprised the entire adult population of Finland. Data collection for the Finnish NHDR is mandatory for all hospitals, including public and private institutions. Data from the NHDR shows good validity regarding both coverage and accuracy [14–16]. Since the data cover the entire country, including both public and private hospitals, the observed changes in rotator cuff repair incidence are representative of nationwide clinical practice and trends for all actively practicing orthopedic surgeons in Finland. During the study period, there were no changes in the known prevalence of rotator cuff disease, the surgical procedure coding system, or the national hospital discharge registry that could explain the entire observed trend. The available data enabled us to also investigate the concomitant procedures associated with rotator cuff repair.

A limitation of this retrospective registry study is that we are unable to evaluate potential inaccuracies in coding of the diagnoses or procedures. Although the accuracy and coverage of NHDR has been found to be excellent (90–95 %) regarding hip and knee diseases, specific information about shoulder disorders does not exist [14–16]. However, we assume that coverage and accuracy do not differ from what is seen in knee and hip. Another limitation of our study is that the diagnosis and procedure coding used during the study period did not allow differentiation between open and arthroscopic procedures, or between partial and full-thickness tears. The registry data also did not provide information regarding the prevalence or incidence of rotator cuff disorders during the study period. Furthermore, we are unaware of potential changes in percentage of patients with private health care insurance. The registry data does not reveal whether there were any changes in the numbers of patients who sought treatment at public and private health care organizations. It is possible that more patients sought treatment from private health care in 2011 compared to in 1998, thus explaining the increased incidence of rotator cuff repair seen in private hospitals.

Rotator cuff tear is most commonly attributed to degeneration of the rotator cuff tendons [2, 3]. Our findings suggested that diagnosis codes were equally distributed between traumatic and non-traumatic etiology of rotator cuff tear. However, these data must be interpreted with caution because it is not always possible to clinically differentiate between a degenerative and traumatic tear. The patient’s and operating surgeon’s interpretation is likely to overestimate the role of trauma in tear development if significant degeneration was also present in a tendon that ruptured after a trauma. A tear could already exist in a tendon but only become symptomatic after trauma. Accident insurance compensation is usually available only due to a traumatic tear, which also may lead to overestimation of the role of trauma.

### Relation to other studies

Other studies have reported increased incidences of rotator cuff repair in selected patient cohorts. Colvin et al. reported a 115 % increase in the incidence of tendon repair, from 41 per 10^5^ individuals in 1996 to 98 per 10^5^ individuals in 2006 in the United States [11]. An English study found that the rate of rotator cuff repair rose from 1.4 per 10^5^ in 2000 to 16.3 per 10^5^ in 2010 in NHS hospitals across England [12]. These studies each investigated highly selected samples. The present study is the first to report true nationwide incidence.

The incidence rate in Finland (from 44 to 131 per 10^5^ adults per year) is higher compared to rates reported elsewhere. Potential explanations for this increasing trend are the growing awareness of rotator cuff disorders, the increased availability of diagnostic (i.e. radiological imaging) methods and arthroscopic surgery, and advancements in surgical techniques. Furthermore, surgical variation also occurs due to differences in physician beliefs regarding indications for surgery, and due to the extent to which patient preferences are incorporated into treatment decisions. Smaller degrees of variability in surgery rates are due to differences in illness burden, diagnostic practices, and patient attitudes [17]. Here we calculated the incidence rates only for the population aged 18 years or older. If the entire population had been included, the incidence rates would be approximately 20 % lower, ranging from 36 to 105 per 10^5^ person-years. However, it was reasonable to exclude individuals younger than 18 years because this population has a very low incidence of rotator cuff repair.

### Clinical and societal implications

Kuhn et al. showed that non-traumatic full-thickness rotator cuff tears can be effectively treated with specific physical therapy in a majority of patients [7]. Moosmayer et al. reported that rotator cuff repair produced significantly better results compared to physiotherapy in a study including both traumatic and non-traumatic tendon tears, based on Constant score (13.0-point difference) and pain measured by visual analog scale (1.7-cm difference) after one year [18]. However, after five years, the differing results between rotator cuff repair and physiotherapy with optional tendon repair levelled out, showing mean between-group differences of 5.3 points in Constant score and 1.1 cm on the visual analog scale for pain, which may not reach clinical importance [19]. Kukkonen et al. performed a study including only non-traumatic rotator cuff tears, and found equal one-year results for rotator cuff repair and non-operative treatment [20]. Overall, the current research suggests that operative treatment of small non-traumatic rotator cuff tears may not be more effective than non-operative management, and thus physicians should prefer a non-operative approach [20].

While rotator cuff repairs are performed with increasing incidence in Finland, the opposite is true regarding the treatment of rotator cuff disease using acromioplasty without rotator cuff repair. Paloneva et al. reported a declining trend in the incidence of acromioplasty without tendon repair, from 163 to 131 operations per 10^5^ between 2007 and 2011. Incidences of rotator cuff repair and acromioplasty without repair were similar in 2011 [21].

Acromioplasty has been used in combination with rotator cuff repair to improve the tendon repair integrity, due to acromial morphology (curved or hooked types), or to make the repair technically easier. Our present data showed that rotator cuff repair was associated with acromioplasty in 40 % of the cases in 2011, and the trend seems to be slightly upwards over the studied time period. However, similar cuff repair results have been found regardless of whether acromioplasty is performed [22, 23]. Regarding the observed rising trend of acromioplasty associated with tendon repair, there is a risk of bias because acromioplasty has previously been considered an integral part of the rotator cuff repair procedure and may not have been recorded using a separate procedure code. Altogether, it appears that there is obvious potential to reduce the operative time and potentially the cost of surgery by avoiding unnecessary resection of the acromion during rotator cuff repair. During the study period, we also noted remarkable increases in the proportions of other concomitant procedures.

In Finland, public health care is provided by the community and is available for every person at a very low cost. People are also free to use private health care, for which the costs are paid partly by government, insurance companies, employers, and/or by the patients themselves. Here we observed a remarkable upward trend in the incidence of rotator cuff repair performed at private health care organizations, whereas the incidence in public hospitals increased until 2005 and remained approximately at the same level thereafter. One explanation for these contrasting trends is that there may have been a change in the proportion of patients seeking treatment for shoulder problems in private hospitals instead of public hospitals. If more patients seek treatment at private orthopedic clinics, the absolute number of operations is likely to rise even with no changes in the indications for surgery.

## Conclusions

The present study showed that the incidence of rotator cuff repair in Finland is rising rapidly, especially in private hospitals. The justification of this progress merits further investigation, because operative treatment of rotator cuff tear has not been shown to be more effective than non-operative management, especially in small non-traumatic tears.
